# Facilitators and Barriers to the Sustainability of eHealth Solutions in Low- and Middle-Income Countries: Descriptive Exploratory Study

**DOI:** 10.2196/41487

**Published:** 2023-05-12

**Authors:** Adane Mamuye, Araya Mesfin Nigatu, Moges Asressie Chanyalew, Lamia Ben Amor, Sihem Loukil, Chris Moyo, Samuel Quarshie, Konstantinos Antypas, Binyam Tilahun

**Affiliations:** 1 College of Informatics University of Gondar Gondar Ethiopia; 2 College of Medicine and Health Sciences University of Gondar Gondar Ethiopia; 3 Amhara Regional Health Bureau Bahir Dar Ethiopia; 4 Sfax HealthTECH Cluster Sakiet Ezzit Tunisia; 5 Health Information Systems Programme Malawi Lilongwe Malawi; 6 Ghana Health Service Accra Ghana; 7 SINTEF Digital Oslo Norway

**Keywords:** attributes, Africa, eHealth, low- and middle-income countries, sustainability

## Abstract

**Background:**

Despite the widely anticipated benefits of eHealth technologies in enhancing health care service delivery, the sustainable usage of eHealth in transitional countries remains low. There is limited evidence supporting the low sustainable adoption of eHealth in low- and middle-income countries.

**Objective:**

The aim of this study was to explore the facilitators and barriers to the sustainable use of eHealth solutions in low- and middle-income nations.

**Methods:**

A qualitative descriptive exploratory study was conducted in 4 African nations from September to December 2021. A semistructured interview guide was used to collect the data. Data were audio-recorded and transcribed from the local to the English language verbatim, and the audio data were transcribed. On the basis of the information gathered, we assigned codes to the data, searched for conceptual patterns, and created emerging themes. Data were analyzed thematically using OpenCode software.

**Results:**

A total of 49 key informant interviews (10 from Tunisia, 15 from Ethiopia, 13 from Ghana, and 11 from Malawi) were conducted. About 40.8% (20/49) of the study participants were between the ages of 26 and 35 years; 73.5% (36/49) of them were male participants; and 71.4% (35/49) of them had a master’s degree or higher in their educational background. Additionally, the study participants' work experience ranged from 2 to 35 years. Based on the data we gathered, we identified 5 themes: organizational, technology and technological infrastructure, human factors, economy or funding, and policy and regulations.

**Conclusions:**

This study explores potential facilitators and barriers to long-term eHealth solution implementation. Addressing barriers early in the implementation process can aid in the development of eHealth solutions that will better fulfill the demands of end users. Therefore, focusing on potential challenges would enhance the sustainability of eHealth solutions in low- and middle-income countries.

## Introduction

The advent of eHealth solutions demonstrated the capability of information and communication technologies (ICTs) to improve health and the health care system. By the definition of Lewis et al [1]*,* “eHealth is the cost-effective and secure use of information communication technology in support of health and health-related fields, including health-care services, health surveillance, health literature, and health education, knowledge, and research.” eHealth includes clinical, administrative, and research-oriented areas [2]. It has been well stated that eHealth solutions contribute to more effective and efficient health care services by improving diagnosis accuracy, optimizing clinical pathways, avoiding duplicate examinations or treatments, and fostering collaboration among health care stakeholders [3]. The World Health Organization's 58th assembly urged countries to develop ICTs for health as deemed appropriate to promote equitable, affordable, and universal access to their benefits [4]. However, eHealth requires the use of appropriate hardware, software, and connectivity technologies for its successful use [5].

Digital health should be an integral part of health priorities, benefiting people in ways that are ethical, safe, secure, dependable, equitable, and sustainable. Transparency, accessibility, scalability, replicability, interoperability, privacy, security, and confidentiality should all be considered when addressing eHealth solutions [6]. The successful use of eHealth technologies is thought to improve health care service delivery by improving health care data management, lowering costs, and decreasing medical errors [7,8]. The long-term sustainability of eHealth technology is dependent on the economic, social, and organizational attributes in which the technology is embedded [9]. Sustainability is defined as the capacity to maintain or improve the state and availability of desirable materials or conditions over a long period of time. It is a normative and fuzzy concept that is determined by the users’ preferences [10]. Similarly, sustainable infrastructure refers to infrastructure projects that are planned, designed, constructed, operated, and decommissioned in a manner that ensures economic and financial, social, environmental, and institutional sustainability throughout the project’s entire life cycle [11].

eHealth technology is regarded as a very useful technological approach to address many challenges related to disease burden, scarcity of health care professionals, inequity in health care service delivery, quality of service delivery, timely decision-making, enabling self-management, medical error, employee and patient satisfaction, health care efficiency, risk analysis, proactive intervention, and inadequate health care budget [9,12-14]. Although a widely anticipated benefit of eHealth technologies in improving the whole health care service delivery outweighs the challenges [15], its sustainability in developing countries is still low [16-18]. This could be due to the limited funding for medical technology, limitation in technological access, low technological literacy, low levels of education, and poor infrastructure [19,20].

A scoping review categorized the challenges for national eHealth system implementation into 5 categories, namely implementation, legal and ethical, data related, engagement, and software related [15]. Another study done in Malaysia also demonstrated that the availability of strong fundamental knowledge, infrastructure, planning and management of health information and technology, fulfillment of legal and ethical issues, and continuous evaluation are all related to the success of telemedicine implementation [21].

Moreover, investment in ICT in many African countries is also limited and has the lowest development index for its successful use [22,23] that could have contributed to the low adoption of eHealth. On top of the limited ICT infrastructure, the financing strategies at the regional and national levels are also found to be inadequate affecting the sustainability of eHealth implementation [24].

Although there is evidence regarding factors that affect successful eHealth implementation [25-29], none of them did explore the facilitators and barriers by involving participants from program implementers, eHealth solution developers, government officials (from ministries, regional, or county leaders), facility administrators, and service providers in resource-constrained countries. The information generated from the diversified participants will help to generate synthesized information and ultimately enable decision-makers to take an informed decision. Therefore, this study aimed to explore the facilitators and barriers affecting the sustainability of eHealth solutions in low- and middle-income countries (LMICs) by including eligible key informants from the aforementioned categories.

## Methods

### Study Design, Participants, and Data Collection

A qualitative descriptive exploratory study was conducted from September to December 2021 in 4 LMICs, namely Ethiopia, Tunisia, Malawi, and Ghana.

In this study, we used current position, proximity to eHealth technology, and work experiences related to eHealth that were considered as criteria to select study participants. Participants from program implementers at various levels, eHealth solutions developers, government officials (ministries, regional, or county leaders), facility administrators, and service providers were included. Thus, 49 study participants were identified purposively using maximum variation techniques in the 4 countries to collect data from as many different perspectives by considering geographical location, organization type, administration role, and service providers.

The total sample size, however, was determined based on information saturation, and data were collected via a face-to-face interview using a semistructured interview guide with multiple probes. Prior communication via phone call and email was carried out to fix a convenient date and time before physically visiting the study participants’ organization.

The key informant interview guide was developed by the research team using published studies [9] and adapted to local contexts by considering the cultural beliefs of the study area. We recruited experienced data collectors and trained them on how to use the interview guide. We examined the tool's content and comprehensiveness to determine whether it would address the research questions. All the collected data were tape-recorded. The average minimum and maximum times of the interviews were 42.5 and 116.75 minutes, respectively. Field notes were also taken to supplement the audio recording.

### Data Analysis

The recorded data were transcribed verbatim and returned to the interviewee for feedback. The transcription was translated into English from native languages by the authors. To ensure proper transcription and translation, the translated data were cross-checked with the audio-recorded file. The transcriptions were coded with Open Code 4.02 software. Line-by-line coding was used to find related patterns. The codes with similar patterns were then combined to uncover themes in the data. Multiple readings were carried out in order to grasp the overall meaning of the data. Additionally, a line number was given for each sentence, and codes were created in order to identify patterns of ideas. The data were scoured for notable patterns and common themes. Themes were thus developed based on emergent ideas. We used thematic analysis using OpenCode software [30].

### Trustworthiness

We reviewed the interview guide with subject matter experts to ensure the accuracy of the data in order to demonstrate the study's validity. The interview guide underwent a pilot test to ensure clarity and the flow of questions. The interviews were administered in the local language and translated into English by the investigators. The researchers spent a lot of time observing and interacting with the study participants. Furthermore, member verification was used both during and after data collection to confirm the details acquired and the interpretation of our findings, ensuring the validity of the data.

### Ethics Approval

The study was carried out in accordance with the World Health Organization Declaration on Ethical Principles [31]. Besides, we have received ethical approval from the institutional review board from the respective participating countries (University of Gondar, Ethiopia, reference number VP/RCS/05/1383/2021; Ministry of Health, Malawi, reference number QMDH/1/1; the Ghana Health Service Ethics Review Committee, Ghana, reference number GHS-ERC: 014/06/21; and the National Insurance for Personal Data Collection, Tunisia, reference number 21/01-1245) not only for its ethical appropriateness but also for methodological soundness. Informed written consent was taken from the study participants, and the information was gathered anonymously with no personal identifiers. To refer to the direct quotations, nonpersonal identifiers were used.

## Results

### Participants’ Demographic Characteristics

A total of 49 key informant interviews were conducted as part of the study (10 from Tunisia, 15 from Ethiopia, 13 from Ghana, and 11 from Malawi). Of these participants, 40.8% (20/49) of them were between the ages of 26 and 35 years, 73.5% (36/49) of them were male participants, and 71.4% (35/49) of them have MSc or above. Furthermore, the work experiences of the participants ranged from 2 to 35 years (Multimedia Appendix 1).

### Theme Formation

We identified 5 themes that are related to human factors, economy or funding, organizational factors, technology and technological infrastructures, and policy and regulations.

#### Theme 1: Human Factors

User-related attributes such as being younger, good awareness, exposure to digital literacy, and commitment were identified as facilitators for the sustainable use of eHealth solutions. This was resounded by another participant:

Those in the older age brackets are less likely to use it compared to the younger one. They pushed their tablet to the young Nexus to demonstrate and I asked them whether they were not part of their training. So the young ones should use and demonstrate to me how the system works.

Male, PG03

Additionally, having a higher education level, positive attitudes, willingness, prior exposure, and acceptability of the eHealth solution by end users was identified as major contributors to the long-term use of digital health applications. Participants in the study also stated that incentivized employees were more likely to use eHealth solutions than nonincentivized ones.

We also observed that high level healthcare professionals have positive attitude towards eHealth solutions implementation. But every time we go down, there are times when health workers did not understand the benefits of implementing eHealth solutions instead end users were reluctant and negligent to use data produced by the eHealth solutions.

Male, PE14

Even though many attributes have been mentioned by study participants as contributing to the sustainable use of eHealth solutions, there are a few that may negatively impact technology's viability. Lack of trained manpower, low digital literacy, lack of commitment and motivation, skill gap, a lack of trust in technology, resistance to change, a negative attitude, and fear of technology were mentioned as potential barriers to the sustainability of eHealth solutions in health care. This idea was expressed by

No one maintains our medical technologies like CT Scan when stop working, but are maintained by someone who comes from other areas, which are the sources of our threat and difficult to trust the care organization that provides the technology since they don’t want to give full responsibility for us.

Male, PE10

#### Theme 2: Economy or Funding

According to the findings, adequate budget allocation, affordability, and profitability of technologies were key enablers of the long-term sustainability of eHealth solutions. This idea was echoed by another participant:

The availability of sustainable funding and solid sponsoring are major facilitators for the implementation of sustainable eHealth solutions. Sufficient budget should cover the implementation of appropriate ICT infrastructure including software, equipment, network, maintenance, updating and end users training.

Female, PT09

However, we identified that lack of adequate funding, high startup cost, and reliance on donors were the major barriers to the sustainability of eHealth solutions.

…it is difficult to allocate sufficient budget to invest in the implementation of eHealth solutions. This always depends on external funding because we don't have a clear eHealth strategy and we don't set aside enough money and funds for this.

Male, PT02

Another participant also added:

…due to the high budget demanding nature, we reached only 50% electronic community health information system health centers coverage at one region of the country.

Male, PE13

#### Theme 3: Organizational Factors

The availability of adequate human resources, technical personnel, information technology experts, and skill training were all significant facilitators of the sustainable use of eHealth solutions. Furthermore, the availability of partners' involvement to cover financial issues as well as the availability of capacity-building activities such as supportive supervision, mentorship, and conducting review meetings was identified as potential enablers for the use of sustainable eHealth solutions. Additionally, having ongoing conversations with staff members, developing a sense of ownership, creating a supportive environment, and encouraging end user participation were noted as crucial facilitators. Organizations with material and management support, leadership engagement, necessary infrastructure, and good workflow were also among the facilitators. We also discovered that the availability of leaders' support, organizational readiness, and organizational structure was reported as reliable facilitators of sustainable eHealth solutions. This was resounded by another participant:

The eHealth solutions don't exist independently. It is the organizations that provide the eHealth solutions. The call managers or the topmost hierarchy of the organization must see health as a priority. Then that would trickle down to the ordinary worker.

Male, PG02

Contrarily, lack of manpower, staff turnover, lack of technical support, lack of digital literacy, and insufficient capacity-building activities were identified as critical challenges to the sustainability of eHealth solutions. Besides, participants added that lack of home-grown systems, lack of system integration, delayed bidding and procurement processes, lack of leadership support, and lack of incentives as major barriers to sustainable use of eHealth solutions.

It is good to use and integrate open sources with our homegrown software. It is dangerous to rely on open source alone because one day when things go the wrong way, it can lead to a devastating outcome. So in-house developed eHealth solutions are more trustable than open-source and partner-based systems.

Male, PE11

#### Theme 4: Technology and Technological Infrastructures

System user-friendliness, system ownership, availability of strong data security, availability of data privacy, and confidentiality of patient data were all mentioned as important factors in the success of the eHealth solution. Furthermore, the usefulness, user-friendliness, and quality of digital solutions have all been identified as facilitators of the sustainable use of eHealth solutions. In addition, reliable internet and local area network connectivity, backup and recovery tools, and larger storage and memory capacity of computers were reported as technology-related facilitators for the sustainable use of eHealth solutions. Furthermore, adequate power backups, adequate electricity (sufficient amount of power to operate the digital imaging machines), and infrastructure accessibility were described as prerequisites related to infrastructure for the long-term use of eHealth solutions. Among the many barriers, poor data security and privacy, software complexity, poor information exchange among users and systems, and lack of system maintenance were frequently mentioned as technology-related barriers. Furthermore, digital solutions that are not adaptable to local contexts were identified as a significant barrier. Likewise, lack of infrastructure, limited capacity of ICT infrastructure, frequent power outages, poor electrical installation, and poor internet connectivity are all potential barriers to the long-term viability of eHealth solutions. The idea was reverberated by

I do not trust the sustainability and trustability of the existing infrastructure (frequent interruption and poor electric installation, no internet connectivity, etc.) to implement eHealth solutions. It needs more strengthening and improving.

Female, PT09

#### Theme 5: Policy and Regulation

The availability of system governance and legislation were identified as key contributors to the sustainability of eHealth solutions. However, lack of policy and legislation and lack of standards were identified as potential barriers to the sustainability of eHealth solutions. This idea was echoed by another participant:

When there are no well documented, sequential rules and tasks. There could be a barrier to eHealth solutions. The right workflow process that would help and definitely seem to move towards its achievements with regards to eHealth solutions.

Male, PG12

On the other hand, participants reported that the availability of policy and legislative frameworks could play a major facilitating role in the sustainability of eHealth solutions:

A regulatory framework for compliance with structures, processes and procedures to guide end users, data security and data exchange standards and laws and guidelines on how to operate the laws in the context of eHealth are very essential.

Male, PT08

However, the participant indicated that due to the lack of policy and legislation system handover was so difficult after the developer:

We don’t trust software offered by external sources because, when they become disappointed at some point, they could demolish the system. When they left the country, they didn’t offer the source code of e-HMIS, EMR, and e-HRIS on which our healthcare system relied.

Male, PE11

## Discussion

### Principal Findings

In this study, we identified the facilities and barriers into 5 themes, namely human, economy or funding, organizational, technology and technological infrastructures, and policy and regulations. The varieties of the themes identified indicate that the sustainability of eHealth solutions is a complex topic. In addition, the themes identified are often closely connected. For example, the theme of the economy is also related to issues mentioned in human resources and infrastructure. End user’s attitudes, shortage of technical experts low digital literacy, poor ICT infrastructure, and technological complexity were all important predictors of the feasibility of eHealth solutions. Moreover, inadequate funding, a lack of organizational support, and a lack of laws and regulations were also reported as key barriers.

This study confirmed that end users’ opinions influenced the use of new eHealth solutions in health care settings. This observation is consistent with previous research findings indicating that end users’ negative attitudes and fears about accepting and using new technologies impede the long-term viability of eHealth solutions [32], despite health professionals' negative perceptions of eHealth solutions gradually decreasing [33].

In this study, the lack of technical experts with the necessary skills and training was identified as a major barrier to sustain the use of eHealth solutions. Although the role of digital illiteracy has been identified more than a decade ago [33], it is still an important inhibitor to the sustainability of eHealth solutions [20]. In the literature, it is also reported that many health professionals still lack the necessary information technology skills to use eHealth solutions [34]. Scholars also recognized that the lack of qualified health personnel rather than technology was the limiting factor [5]. This implies that capacitating users through training and frequent support is essential for establishing a stable working environment [17,35].

Economy and financing issues are also related to the workforce shortages in health care that also affect the sustainability of eHealth solutions. Our research found that the initial cost of developing and deploying eHealth solutions was prohibitively high, negatively impacting eHealth technology implementation. This finding is also supported by the literature, indicating that long-term financing sources are very crucial for the adoption and successful implementation of new eHealth technologies [1,36]. This implies that shortage of funds would make eHealth solutions implementation difficult.

Previous studies have also indicated that high telecommunication costs and end user training expenses are all common challenges, particularly for developing countries [21,26,35]. As a result, the majority of eHealth systems rely on external funding to assist them to overcome budgetary constraints [9,37]. However, given the limited lifespan of donor-driven eHealth solutions, their long-term viability is still in doubt. A previous study also suggested that the ability of the technology to be adapted to meet the local environment is an essential element in vendor and technology selection [38]. This indicates that employing home-grown eHealth solutions is preferable for the sustainability of eHealth solutions.

The important role of the economy theme is confirming the existing literature, and it seems to be a common issue both in high-income countries and LMICs [24]. A qualitative study conducted in Malawi [39] indicated that stable funding is a very crucial aspect of improving eHealth sustainability. As it has been mentioned by our informants, the financing often comes from donors, and this is believed to further challenge sustainability because donor funding might be short-term or not comply with the country's priorities and needs. National funding needs also to be strategic and long-term oriented; otherwise, it will impose challenges for the long-term viability of the system [24,39]. The economy can also be related to the infrastructure problems that LMCIs face when trying to avail adequate infrastructure specifically for eHealth solutions sustainability and scale-up [17,27]. Key informants reported that organizational support is essential to sustain eHealth solutions. The finding of the study is congruent with previous studies [39,40]. This implies that if the organization is not supporting the end users by providing either on-site or off-site training and incentivizing top performers the sustainability of the eHealth solutions is questionable.

In this study, the complexity of the technology was reported as a barrier to the feasibility of eHealth solutions. In addition to the complexity of the technology, the limited access to network infrastructure was also reflected as a critical barrier to eHealth solution adoption. This finding is in agreement with previous studies [17,19,41]. This implies that the availability of adequate ICT infrastructure and the user-friendliness of eHealth solutions are equally important for its sustainability.

Previous studies revealed that analyzing the status of regulations at the time of eHealth implementation is important for eHealth solution sustainability [21,36,42]. Maintaining patient safety and privacy policies would also significantly contribute to the sustainability of eHealth solutions [15,43]. In our study findings, a lack of policies and legislation was noticed in all the study areas. Moreover, the finding of the study indicated that legal and ethical challenges are essential to successfully implement eHealth solutions. This implies that developing and endorsing policies and standards related to eHealth solutions at the national level is required to promote the acceptance and sustainability of eHealth. Despite the many challenges related to sustainability for eHealth that we identified, there are also promising approaches for improving sustainability. The scarcity of human resources is a challenge, but it can also be a factor in success. Good eHealth has the potential to increase the efficiency of existing health care workers, improve the quality of the services delivered, and increase their accessibility. The economic issues might be challenging the ability of donors and governments to invest in expensive technology, but this increases the chances of investments in home-grown, open-source solutions that are adapted to the local infrastructure. These solutions might be easier and cheaper to maintain over time, have easier access to local user support, and improve the capacity and competence of the national eHealth market. At the same time, policies should ensure that investments have a long-term orientation and conform to standards for security, privacy, and interoperability.

### Limitations of the Study

As a limitation, the study included participants only from the 4 African countries which might have led to the underrepresentation of potential eHealth experts. However, to minimize the bias, we have tried to include domain experts from different sectors and considering their current position, knowledge in the field of eHealth, and their related work experience. This study also has the limitation of not analyzing the efficacy and cost-effectiveness of using eHealth.

### Conclusions

In conclusion, although eHealth has been identified as a promising area of innovation for addressing health system challenges, LMICs continue to face many obstacles to implement new eHealth solutions. As a result, identifying potential facilitators and barriers to eHealth implementation in LMICs is crucial for program implementers and policy makers. Our qualitative study found that lack of adequate infrastructure, shortage of budget, and shortage of technical expertise are among the top challenges in implementing and sustaining eHealth solutions. The study also indicated that donor-driven eHealth solutions face sustainability challenges. This implies that context-based home-grown eHealth solutions are perceived as more useful for the sustainability of eHealth solutions. These findings imply that top-level managers shall give attention to address challenges related to organizational barriers, infrastructure, and manpower. We suggest future research by increasing the number of representative countries with other mixed data collection methods such as focus groups and observation to explore facilitating and inhibiting factors in more detail.

## Appendix

Multimedia Appendix 1 Demographic characteristics of the participants.

## Abbreviations

ICT: information communication technology
LMIC: low and lower-middle-income countries

## Data Availability

This manuscript contains all data produced or examined during this study. We do not intend to share the data because this is a qualitative study with participant identifications in the raw data. However, data can be made available upon reasonable request from the corresponding author.
