# Supplementary material for: Facilitators and Barriers to the Sustainability of eHealth Solutions in Low- and Middle-Income Countries: Descriptive Exploratory Study
Source: JMIR Form Res. 2023 May 12;7:e41487. doi: 10.2196/41487 (PMC10221492; doi:10.2196/41487)
Supplement: Multimedia Appendix 1 [file formative_v7i1e41487_app1.docx]

**Appendix A:** Demographic characteristics of participants

| Participant ID | Age group  (in year) | Sex | Level of education | Experience (in years) |
| --- | --- | --- | --- | --- |
| PT01 | 46 and above | Male | PhD | 26 |
| PT02 | 46 and above | Male | PhD | 20 |
| PT03 | 36-45 | Female | PhD | 5 |
| PT04 | 46 and above | Female | PhD | 35 |
| PT05 | 46 and above | Male | PhD | - |
| PT06 | 36-45 | Male | MSc | 7 |
| PT07 | 46 and above | Male | PhD | 30 |
| PT08 | 46 and above | Male | PhD | 16 |
| PT09 | 18-29 | Female | Diploma | 2 |
| PT10 | 36-45 | Female | PhD | 5 |
| PE01 | 36-45 | Male | MBA | 14 |
| PE02 | 26-35 | Male | BSc. | 12 |
| PE03 | 36-45 | Male | MSc. | 15 |
| PE04 | 36-45 | Female | BSc. | 18 |
| PE05 | 26-35 | Male | MSc. | 10 |
| PE06 | 26-35 | Male | BSc | 4 |
| PE07 | 26-35 | Female | MPH | 7 |
| PE08 | 36-45 | Male | MSc | 12 |
| PE09 | 26-35 | Male | MSc | 10 |
| PE10 | 26-35 | Male | MPH | 12 |
| PE11 | 26-35 | Male | BSc | 9 |
| PE12 | 26-35 | Female | MSc | 10 |
| PE13 | 36-45 | Male | MSc | 14 |
| PE14 | 26-35 | Female | BSc | 6 |
| PE15 | 36-45 | Male | MSc | 20 |
| PG01 | 36-45 | Male | Tertiary | 10 |
| PG02 | 36-45 | Male | Tertiary | 15 |
| PG03 | 36-45 | Male | MSc | 16 |
| PG04 | 26-35 | Male | Tertiary | 7 |
| PG05 | 26-35 | Male | Tertiary | 10 |
| PG06 | 26-35 | Male | Tertiary | 10 |
| PG07 | 26-35 | Male | Tertiary | 7 |
| PG08 | 46 years and above | Male | Tertiary | 20 |
| PG09 | 18-29 | Male | Tertiary | 7 |
| PG10 | 46 and above | Male | Diploma | 23 |
| PG11 | 46 and above | Male | MSc | 7 |
| PG12 | 36-45 | Male | BSc | 11 |
| PG13 | 36-45 | Male | PhD | >15 |
| PM01 | 26-35 | Male | BSc. | 8 |
| PM02 | 36-45 | Male | MSc. | 15 |
| PM03 | 26-35 | Male | Diploma | 9 |
| PM04 | 36-45 | Female | MSc. | 18 |
| PM05 | 26-35 | Female | MSc. | 4 |
| PM06 | 36-45 | Female | MSc. | 14 |
| PM07 | 36-45 | Female | MSc. | 17 |
| PM08 | 36-45 | Male | BSc. | 16 |
| PM09 | 26-35 | Male | MSc. | 5 |
| PM10 | 46 and above | Male | MSc. | 28 |
| PM11 | 18 – 25 | Female | Diploma | 9 |
| PM12 | 15 | Female | Not identified | 27 |

PT: Participant from Tunisia; MSc: Masters of Science; BSc: Bachelor of Science; PhD: Doctor of Philosophy; MPH: Master of Public Health; MBA: Master of Bachelor of Arts

**Appendix B:** Interview time elapsed by country

| No | Country | Minimum time elapsed for a single interview  (in minutes) | Maximum time elapsed for a single interview (in minutes) | Average time elapsed  (in minutes) |
| --- | --- | --- | --- | --- |
| 1 | Tunisia | 35 | 121 | 83 |
| 2 | Ghana | 90 | 150 | 120 |
| 3 | Ethiopia | 21 | 76 | 40 |
| 4 | Malawi | 24 | 120 | 45 |
| Overall Average | | **42.5** | **116.75** | **72** |
